# Social isolation disrupts hippocampal neurogenesis in young non-human primates

**DOI:** 10.3389/fnins.2014.00045

**Published:** 2014-03-27

**Authors:** Simone M. Cinini, Gabriela F. Barnabe, Nicole Galvão-Coelho, Magda A. de Medeiros, Patrícia Perez-Mendes, Maria B. C. Sousa, Luciene Covolan, Luiz E. Mello

**Affiliations:** ^1^Departamento de Fisiologia, Universidade Federal de São PauloSão Paulo, Brazil; ^2^Departamento de Fisiologia, Universidade Federal do Rio Grande do NorteNatal, Brazil; ^3^Departamento de Ciências Fisiológicas, Universidade Federal Rural do Rio de JaneiroSeropédica, Rio de Janeiro Brazil

**Keywords:** social isolation, young marmosets, hippocampal neurogenesis, anxiety, isolation stress

## Abstract

Social relationships are crucial for the development and maintenance of normal behavior in non-human primates. Animals that are raised in isolation develop abnormal patterns of behavior that persist even when they are later reunited with their parents. In rodents, social isolation is a stressful event and is associated with a decrease in hippocampal neurogenesis but considerably less is known about the effects of social isolation in non-human primates during the transition from adolescence to adulthood. To investigate how social isolation affects young marmosets, these were isolated from other members of the colony for 1 or 3 weeks and evaluated for alterations in their behavior and hippocampal cell proliferation. We found that anxiety-related behaviors like scent-marking and locomotor activity increased after social isolation when compared to baseline levels. In agreement, grooming—an indicative of attenuation of tension—was reduced among isolated marmosets. These results were consistent with increased cortisol levels after 1 and 3 weeks of isolation. After social isolation (1 or 3 weeks), reduced proliferation of neural cells in the subgranular zone of dentate granule cell layer was identified and a smaller proportion of BrdU-positive cells underwent neuronal fate (doublecortin labeling). Our data is consistent with the notion that social deprivation during the transition from adolescence to adulthood leads to stress and produces anxiety-like behaviors that in turn might affect neurogenesis and contribute to the deleterious consequences of prolonged stressful conditions.

## Introduction

In the adult hippocampus, progenitor cells in the subgranular zone of the dentate gyrus give rise to new neurons that migrate into the granule cell layer, differentiate into granular neurons, and are capable of functional integration into the hippocampal circuitry (Gould and Gross, 2002; Van Praag et al., 2002; Kee et al., 2007). The functional role of hippocampal neurogenesis has not been fully understood until now, but despite the divergent results from different laboratories and models, most data points toward its involvement with specific aspects of learning, conditioning, and spatial information (for review see Balu and Lucki, 2009).

Reduction in hippocampal neurogenesis is associated with stress (Gould et al., 1998) mainly by means of increased excitatory transmission (Gould et al., 1997; Abraham et al., 1998), pro-inflammatory cytokines (Koo and Duman, 2008), diminished neurotrophins expression (Duman and Monteggia, 2006; Jacobsen and Mork, 2006), and glucocorticoid signaling (Wong and Herbert, 2005, 2006). Social isolation is a form of stress, which affects some hippocampal-related functions such as learning and memory and may lead to affective disorders. In marmosets there is a strong exponential negative correlation between the number of dentate proliferating cells and aging where 2–3 years-old animals are considered young adults, from 4 to 7 years they are middle-aged and above 8 years old they are considered old (Bunk et al., 2011). In the present study we used social isolation of young animals as the stressful event (Laudenslager et al., 1995; Stranahan et al., 2006) in order to characterize behavioral consequences of social isolation during the transition phase from adolescence to adulthood, when the animals are at the peak of dentate neurogenesis, so any disturbance might bear a greater relevance in the onset of future mood disorders.

The long-term effects of social isolation among rodent pups include decreased hippocampal neurogenesis, which can culminate in a reduced ability to cope with stressful events in adulthood (Laudenslager et al., 1995; Mirescu et al., 2004; Karten et al., 2005; Stranahan et al., 2006; Rizzi et al., 2007). As compared to rodents, social interactions in primates are considerably more important for the appropriate neuropsychological development (Rosenblum and Andrews, 1994). Marmosets partially deprived of parental care during infancy develop abnormal patterns of behavior that persist even when they are later reunited with their parents (Dettling et al., 2002a,b). In spite of the well-characterized behavioral consequences of social isolation during infancy in these animals, little is known about the neurobiological effects of social isolation during its transition to adulthood. In the present study we investigate the consequences of social isolation in the behavior and hippocampal neurogenesis in these non-human primates.

## Methods

### Animal care

All experimental procedures were approved by the Research Ethics Board of the Federal University of São Paulo and by the Ethics Committee of the Department of Physiology of Federal University of Rio Grande do Norte. The collected behavioral data was obtained in the animal facilities from both Universities, while the data for brain analysis were obtained from animals raised in Federal University of São Paulo animal facility. The cortisol and behavioral analysis were held at Federal University of Rio Grande do Norte. We are fully aware that under ideal experimental conditions all animals should have been subjected to exactly the same conditions. Here, in order to maximize the availability of animals, to expand the multiple uses of animals and to minimize the unnecessary deaths of non-human primates we opted to use two separate groups of animals under similar laboratory conditions. Indeed, the similarity in the behavioral repertoire of both groups before and after isolation clearly indicates a similar underlying physiological response to social isolation.

Marmosets (*Callithrix sp)* bred in both animal facilities (*n* = 16) were housed in cages, on a standard light/dark cycle (12/12 h). The animals were fed twice a day, around 8:00 am with a protein mixture (powdered milk, corn syrup, eggs, bread, soy bean protein, and bone flour supplemented with vitamins A, D, and E) and around 2:00 pm with a portion of regional tropical fruits. Water was provided *ad libitum*. At baseline, animals were kept with other members of their families (mother, father, and more than one sibling).

In the São Paulo group, 8–10 months old marmosets (*n* = 9) had regular weight (3 males, 198 ± 38 g; 6 females, 223 ± 27 g) in the beginning of the experiments. Animals were subdivided in 3 different groups, each containing 2 females and 1 male. Two groups were socially isolated for 1 or 3 weeks, respectively named here as 1W and 3W groups. Isolated marmosets were kept on individual metallic cages (0.75 × 0.75 × 0.8 m) and had no physical or visual contact with other members of the colony. The third group of animals (control, CTR) remained with their families in metallic cages (1.5 × 1.5 × 0.8 m) for 3 weeks. At the end of the third experimental week all these animals were perfused for immunolabeling of hippocampal proliferating cells.

The Rio Grande do Norte group consisted of 7 animals, with same age and similar weight as the Sao Paulo group (4 males, 200 ± 35 g; 3 females, 220 ± 18 g). Similarly to the São Paulo group, the baseline behavioral repertoire of these sub-adults marmosets was taken for each animal while they were with their families, housed in brick wall cages (2 × 2 × 2 m). Thereafter, animals were subdivided into the social isolated groups 1W and 3W. During isolation marmosets were kept on individual cages (2 × 2 × 1 m) without physical or visual contact with other members of the colony. Finally, after a period of social isolation, marmosets were returned to their family cages and their behavioral repertoire was also assessed to evaluate their familiar reunion.

### Behavioral observation and cortisol measurement

The behavioral repertoire assessment and the feces collection for cortisol levels measurements were made once a day, 2 days prior to the social isolation in order to establish its baseline levels. Feces samples were taken in the first 2 days of social isolation (early phase) and again in the last 2 days (late phase) totalizing four samples per subject during the isolation period. Behavioral observations were also made during the first and last 2 days of the isolation period. The animals were then moved back to their original family cage and again, feces collection and behavioral observations were made during the two following days, considered the reunion phase. The choice of assessment of cortisol levels from collected feces enabled us to avoid the manipulation of animals and the stress related to daily blood sample collection, which would in turn have influenced the animal's behavior.

The fecal collection was performed between 06:30 and 09:00 a.m. to avoid circadian influence on cortisol (Raminelli et al., 2003). All animals were observed until the defecation occurred. After that, a collector entered the cage and collected the feces from the cage bedding on the floor. Only completely identified feces were collected. Material of defecation was collected in small identified glass tubes with snap caps. After collection, the samples were frozen immediately.

Fecal samples were allowed to reach room temperature for at least 10 min before homogenization. Separation of the steroids by extraction was performed on 0.1 g of well-mixed feces by the addition of 2.5 mL ethanol and 2.5 mL distilled water. The samples were vortexed for 5 min and centrifuged for 10 min at 3000 rpm, and then the aqueous phase was decanted into glass tubes and then frozen at −20°C until solvolysis procedure.

Solvolysis was performed as previously described (Ziegler et al., 1995, 1996). To separate the steroid conjugates, frozen samples were left at room temperature for 10 min before the 500 samples were vortexed for 10 s and were added 100 mL solution of sodium chloride (NaCl) saturated, 50 mL sulfuric acid (H_2_SO_4_), and 5 mL of organic solvent ethyl acetate (C_4_H_8_O_2_). The solutions were vortexed for 1 min and kept under agitation in a water bath (40°C) overnight. Next morning, 4 mL of ethyl acetate was added to the mixture; this was followed by 5 min vortex and centrifuged for 3 min (1000 rpm). After supernatant extraction, 2.5 mL of distilled water was added and the samples were vortexed 5 min, followed by 3 min centrifugation at 1000 rpm. The supernatants were allowed to dry on a water bath at 40°C until its complete evaporation. Finally, the fractions of free steroids were ressuspended in 500 μL of ethanol and assayed for cortisol using a modification of the enzyme immunoassay method developed by (Munro and Stabenfeldt, 1984; Ziegler et al., 1995, 1996) with the standards used for this assay prepared in ethanol. Parallelism using serial dilution of marmoset fecal pool and standards did not differ (*P* > 0.05), and the accuracy was 109.6 ± 4.4%. For this, 50 μL of each sample was placed on a water bath 40°C until its complete evaporation. Immediately following was added 300 μL of solution containing the enzyme conjugate (cortisol:HRP, diluted at 1: 75,000) to the respective hormone (dilution 1:16,000 in monophosphate buffer:sodium diphosphate). The solution was kept under agitation for 5 min. Then, 100 μL of each sample was placed in multiwell plates prepared with the cortisol antibody (R4866, 1:12,000), which was developed and characterized by Munro and Stabenfeldt (1984). The samples were incubated for 2 h in a humid chamber. After that, 100 μL/well a solution containing 25 mL of 10% citrate buffer, 250 μ L of ABTS substrate [2,2′-Azino-bis (3- ethylbenzothiazoline-6-sulfonic acid) diammonium salt, Sigma] and 80 μL of H_2_O_2_ was added to each well. Thus, the multiwell plate was incubated for 1 h in humidity chamber. A solution containing 5.048 mL of hydrofluoric acid; 1.2 mL of sodium hydroxide (5N) diluted in 500 mL of distilled water was prepared. From this solution, 100 μL of was added to each well to stop the reaction. The optical density reading of the plaque was assessed with a spectrophotometer (Asys/Hitech Expert plus, Eugendorf, Austria) using 410 nm filter.

The whole behavioral repertoire analysis was performed during 2 h daily using the focal continuous observation. This method has been previously demonstrated to be effective to monitor the bimodal pattern in captive common marmosets (Barbosa and Mota, 2009). For statistical analysis we selected anxiety-related behaviors (Barros and Tomaz, 2002; Dettling et al., 2002a), like scent-marking (frequency of rubbing the anogenital on a surface of cage), scratching, piloerection, and locomotor activity (the cage was divided into 60 quadrants of 38 cm each and the frequency of movement between two quadrants was recorded as one movement), as well as behaviors that are indicative of attenuation of tension like auto-grooming: time spent licking, picking at or parting his or her own fur with the fingers (Devries et al., 2007; Wittig et al., 2008), attempts to establish contact, and social-grooming.

### BrdU and doublecortin staining

On the last day at end of 1 (1W group) or 3 weeks (3W group) of social isolation or 3 weeks after the beginning of the experiments (CTR group), animals (*n* = 3 per group) were given a single injection of 5-bromo-2′-deoxyuridine (BrdU; 75 mg/Kg i.p. dissolved in a solution containing 0.9% NaCl and 0.007 M NaOH). Twenty-four hours later with the animals still in isolation, they were deeply anesthetized with thiopental (50 mg/Kg, i.p) and transcardially perfused with 0.9% saline, followed by a 4% paraformaldehyde fixative solution. The brains were then removed from the skull and post-fixed overnight in the same fixative solution before cryoprotection in 30% sucrose solution. Thirty-two μm thick coronal sections throughout the entire antero-posterior axis of the hippocampus were cut on a cryostat. Sections from each animal were sequentially collected in 24-well plates filled with anti-freezing solution and stored at −20°C.

The immunohistochemistry procedure was a priori defined and it was performed on every 12th section of the entire hippocampus (the distance between each two sections was about 360 μm). With this sampling, we were able to run countings in 1 out of 12 representative sections along the rostro-caudal extension of the dentate gyrus. The tissue was first incubated with 3% H_2_O_2_ in PB (10 min), washed in PB, followed by 2M HCl at 40°C (30 min), and washed in PBS (3 × 10 min). Sections were then incubated for 1 h in a blocking buffer solution containing 2% normal goat serum (Sigma-Aldrich) and 0.1% TritonX-100 in PBS. Thereafter, they were incubated with monoclonal primary antibody to BrdU (rat anti-BrdU, 1:200, Axyll/Accurate Chemical) in the blocking solution overnightat 4°C. In the next morning, sections were washed in PBS and incubated with biotinylated secondary antibody (goat anti-rat IgG, 1:200, Vector) during 2 h in PB. After 3 washes in PBS (3 × 10 min) were incubated for 1 h in avidin-biotin-peroxidase complex (Vectastain Elite ABC,Vector) and the reaction product was developed using 0.05% 3,3′-diaminobenzidine-tetrahydrochloride(DAB, Sigma-Aldrich). Stained sections were mounted onto gelatin-coated glass slides, air dried, dehydrated, and coverslipped with Entellan (Merk).

Co-localization of BrdU and doublecortin (DCX) was examined in the sections adjacent to those used for BrdU labeling. Sections were incubated in HCl 2M at 40°C for 30 min, followed by incubation with a mixture of the monoclonal primary antibodies to BrdU (rat,1:200; Axyll/Accurate Chemical) and DCX (guinea pig, 1:100; Chemicon) diluted in blocking buffer overnight, at room temperature. After several rinses in PBS, sections were incubated with Alexa Fluor® 488 goat anti-rat IgG (1:300, Molecular Probes) and Alexa Fluor® 546 donkey anti-guinea pig IgG (1:300, Molecular Probes) for 1 h at room temperature. Rinsed tissue sections were then mounted onto gelatin-subbed slides and coverslipped with Fluoromount G (Electron Microscopy Sciences).

### Cell counts

Cell counts were conducted on a blinded fashion at 3 different rostro-caudal levels throughout the hippocampal extent of 9 animals. These corresponded to plates A 6.5–A 5.5 (rostral level), A 5.0–A 4.0 (intermediate level) and A3.5–A 1.5 (caudal level) of the marmoset brain atlas by Stephan et al., 1980. Both right and the left hippocampi were assessed, rendering a total of 55, 58, and 30 coronal sections counted in the groups of animals isolated for 1 week, 3 weeks and controls, respectively. Since the number of proliferating cells labeled with BrdU or BrdU/DCX in the dentate gyrus was very low and the spatial distribution was inhomogeneous, the total number of labeled cells was counted in the selected sections, as above described, for each animal. For this, we took care to assure that each analyzed section was similar among all groups.

To quantify BrdU-positive cells, we have used a light microscope (400 × magnification; Olympus® BX 50 microscope, Japan) and a digital image analysis system (Neurozoom software, NY, USA). Small BrdU-labeled nuclei in the hilar border of the dentate gyrus (presumed to be glial precursors) and fusiform immunostained cells (endothelial-like) were excluded from the analysis. In order to normalize data, the number of BrdU-positive cells in the three different groups of animals is expressed as the mean number of labeled cells per section.

The number of cells co-localizing BrdU and DCX was determined with a Zeiss (Oberkochen) LSM 510 confocal laser microscope. Sections were scanned using a 40 × oil-immersion objective and dual-channel excitation with argon (488 nm) and helium-neon (543 nm). Four coronal sections per subject corresponded to the ones described above were randomly assessed for counting. Co-localization analysis included visual inspection of size and shape of cell throughout a z-stack and orthogonal planes. The percentage of co-localization was achieved by dividing the number of double-labeled cells (DCX/BrdU-positive) by the total number of BrdU-positive cells in each hippocampal section.

### Statistical analysis

The comparison of cortisol levels between baseline, early social isolation, late social isolation, and after family's reunion was assessed by means of repeated measure analysis of variance followed by *post-hoc* Fisher PLSD. The variation of behaviors between phases was assessed with non-parametric tests (Friedman, Wilcoxon, Mann-Whitney). Pearson's correlation coefficient (*r*) was obtained to investigate the relationship between anxiety-like behaviors with the cortisol levels along different phases of social isolation. Differences in the total number of BrdU-positive cells between groups, across hippocampal plates or the full structure, were assessed with Two-Way and One-Way ANOVA (applying Newman-Keuls *post-hoc* test), respectively. Percentages of BrdU and DCX co-localization were analyzed by Kruskal-Wallis and Mann-Whitney tests. After the percentage was determined for the total BrdU counting, we performed One-Way analysis of variance followed by Newman-Keuls *post-hoc* test. Statistical significance was set at *P* ≤ 0.05. Results in the text are expressed as mean ± standard error (SE).

## Results

### Different durations of social isolation resulted in behavioral changes among young marmosets

**Figure 1:**
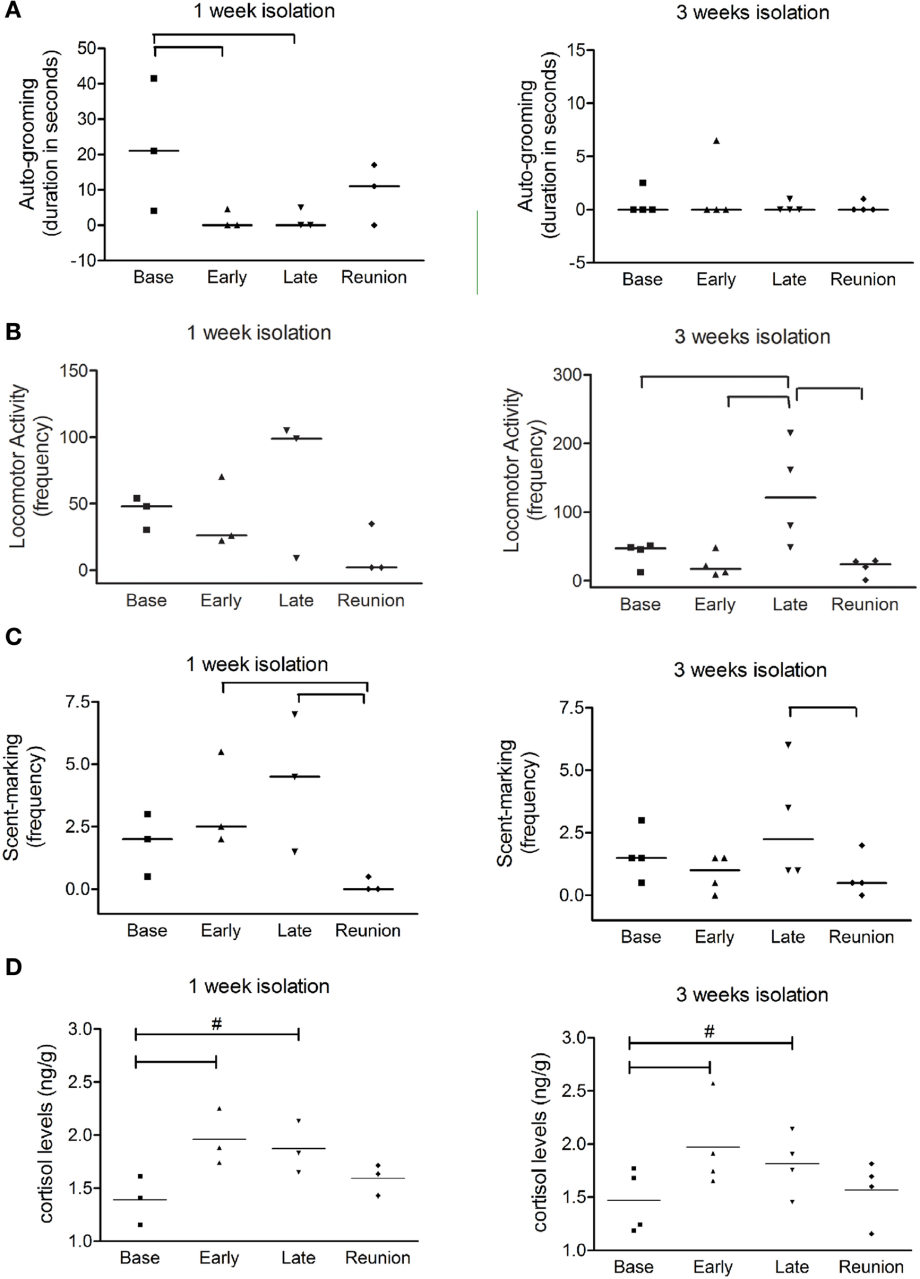
**Behavioral changes produced by isolation and cortisol measurement from fecal samples**. Scatter plot graphs from behavioral observation data show **(A)** duration of auto-grooming (expressed in seconds); **(B)** frequencies of locomotor activity; and **(C)** frequencies of scent-marking during baseline phase (Base), two initial days of isolation (Early), two finals days of isolation (Late) and reunion for both 1 week and 3 weeks social isolation. In **(D)**, scatter plots represent normalized cortisol levels for marmosets isolated during one and 3 weeks; Horizontal lines indicate median values; brackets designate significant alterations between phases (*P* < 0.05). # Indicate tendency of variation between respective phase and baseline phase (0.05 < *P* < 0.06).

Animals that have been isolated for 1 week decreased auto-grooming at both early and late phases of isolation, when compared to baseline values (*X*^2^ = 9.52, *P* = 0.02; Basal × Early phase: *z* = 2.02; *P* = 0.04; Basal × Late phase: *z* = 2.02; *P* = 0.04) (Figure 1A). The 1W group also had higher frequency of scent-marking during both phases of isolation than in the reunion phase (*X*^2^ = 7.8, *P* = 0.05; Reunion × Early phase: *z* = 2.02; *p* = 0.04; Reunion × Late phase: *z* = 2.02; *P* = 0.04) (Figure 1C).

Animals isolated for 3 weeks had higher frequencies of locomotor activity at later phase of isolation than during other phases (*X*^2^ = 13.8, *P* = 0.03; Basal × Late phase: *z* = 2.52; *P* = 0.01; Early × Late phase: *z* = 2.36; *P* = 0.01; Reunion × Late phase: *z* = 2.36; *P* = 0.01) (Figure 1B) and higher frequencies of scent-marking at late phase of isolation than after reunion (*X*^2^ = 8.19, *P* = 0.04; Reunion × Late phase: *z* = 2.02; *P* = 0.04) (Figure 1C). Altogether these data indicate that marmosets had a higher expression of anxiety-related behaviors and a reduction of those related to attenuation of tension during social isolation, but we were not able to distinguish the effects between 1 and 3 week groups. We did not observe any other behavioral alteration regarding the other analyzed parameters, such as piloerection, attempts to contact and social-grooming. It is important to emphasize that all anxiety-related behaviors returned to their basal levels after family reunion, as well as the social grooming, which is an indicative of good adaptation. This result is confirmed by the cortisol levels measurement in the reunion phase, as described below.

### Social isolation induced stress in marmosets

In order to infer about stress levels during social isolation, each session of behavioral monitoring was followed by cortisol levels measurement, for all animals under study (Figure 1D). The early phase of social deprivation caused significant increase in cortisol in both 1W (*P* = 0.03) and 3W groups (*P* < 0.01) when compared to their respective baseline levels. However, thereafter cortisol levels gradually decreased, reaching values between baseline and early isolation phases (*P* = 0.057). After family reunion, the cortisol levels of previously isolated marmosets became similar to their respective baseline values (*P* > 0.05).

Positive correlations were observed between anxiety-related behaviors and cortisol levels indicating that animals had indeed experienced stress. The cortisol level was significantly correlated with the frequencies of scent-marking during the early phase of social deprivation (*r* = 0.53, *P* < 0.05) and with increased locomotion (*r* = 0.10, *P* < 0.05) in the later phase of social deprivation.

### Hippocampal cell proliferation and neurogenesis are impaired in isolated marmosets

**Figure 2:**
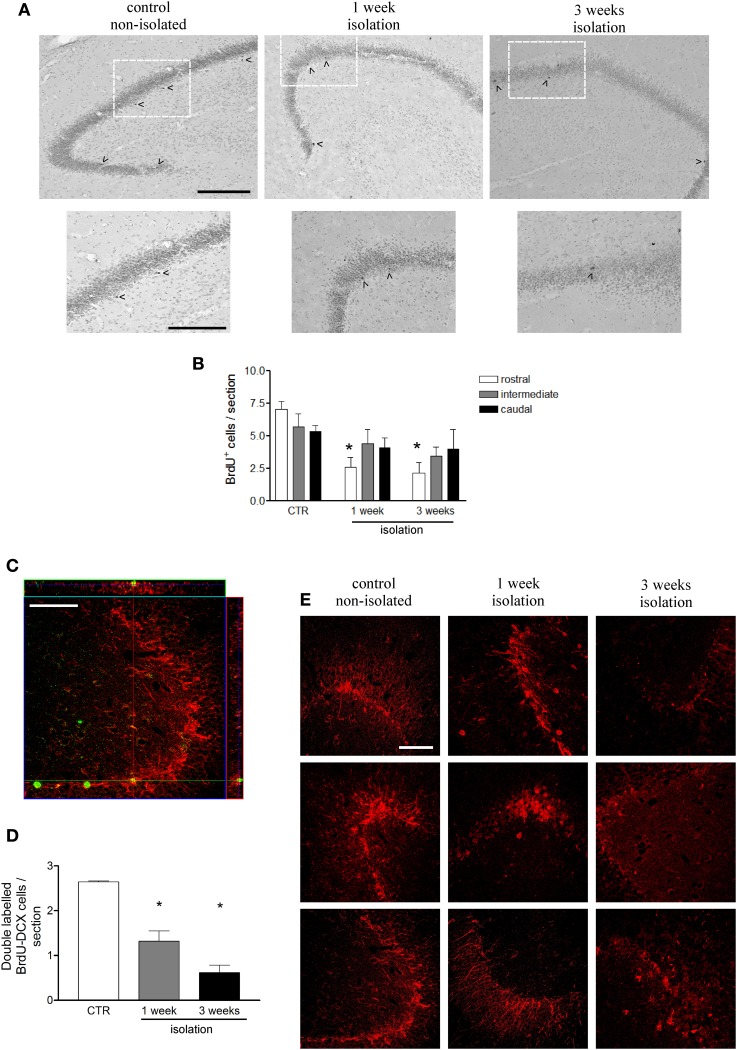
**Effects of social isolation on hippocampal cell proliferation. (A)** Representative images of BrdU-positive cell labeling in dentate gyrus subgranular area in rostral region for each group in a wider view (upper panel) and in detail of the indicated area as showed in the lower panel; arrow heads indicate cells included in the counting; scale bar for upper panel = 200 μm; scale bar for lower panel = 150 μm. **(B)** graphical representation of the total number of BrdU-positive cells per section at three different rostral-caudal levels of the hippocampus for animals that were socially isolated for 1 week, 3 weeks, and non-isolated age matched controls (CTR). **(C)** Orthogonal Z-section of confocal microscope image of BrdU-DCX double-labeled cell in hippocampal dentate gyrus used to estimate the number of new cells undergoing a neuronal fate per section, showed in graph **(D)**. Figure **(E)** represents DCX-immunorreactivity density in dentate gyrus for each marmoset under control or 1 weeks or 3 weeks isolation periods. BrdU labels in green and DCX labels in red; scale bar: 50 μm. Data represented as mean ± standard error. ^*^ Indicates statistical significance between CTR and the respective group.

Cell counts of the entire hippocampus showed that the number of dentate gyrus progenitor cells incorporating BrdU was significantly decreased in animals that were socially isolated for 1 (3.71 ± 0.65 cells/section) or 3 (3.20 ± 0.87 cells/section) weeks, as compared to controls (6.04 ± 0.04 cells/section; *P* = 0.04, *F* = 5.82) (Figure 2A). In order to verify if this reduction was homogeneous throughout the rostro-caudal extent of the hippocampus, we further evaluated the distribution of BrdU-labeled cells in rostral, intermediate and caudal levels of hippocampus, as described in Methods. A One-Way ANOVA indicated that major BrdU-labeled cell reduction occurred in rostral levels, where 1W and 3W groups were statistically different from non-isolated animals (*P* < 0.01, *F* = 14.98). It also revealed that cell proliferation in the intermediate or caudal portions of dentate gyrus was similar between 1W, 3W, or CTR marmosets (Figure 2B). No interaction was found between groups and hippocampal levels (Two-Way ANOVA; *P* = 0.26, *F* = 1.44).

To assess whether newborn cells underwent neuronal fate, sections were double-stained for BrdU and DCX (a marker of immature neurons) (Figure 2D). Among the BrdU-labeled cells 43.7% co-localized to DCX in non-isolated controls, 35.5% in 1W group, and 19.2% in 3W group. These proportions pointed toward a decrease in the neurogenesis rate after 3 weeks isolation (*P* = 0.05, compared to Control and 1W groups). Taken together, these results suggest that marmosets that were socially isolated not only had a reduced number of dentate gyrus proliferating cells, but also fewer of these cells underwent a neuronal phenotype. Thus, once the number of newborn neurons can be estimated by applying the proportion of double-labeled cells over the total BrdU counting (Figure 2C), the neurogenesis impairment among the isolated primates can be clearly appreciated (*P* = 0.0004, *F* = 39.17). The late effects of neurogenesis impairment after 3 weeks of social isolation resulted in an overall reduced DCX immunoreactivity in the dentate gyrus, as illustrated in Figure 2E, which includes a representative image for each subject in all three groups.

## Discussion

In summary, our results led us toward a possible cascade of events from social isolation to hippocampal substrates of anxiety-related behaviors during early adulthood of non-human primates. Here we found that socially isolated young marmosets had an increase in cortisol levels during the first days of isolation, concomitant with changes in affective states and subsequently a steady decrease in hippocampal neurogenesis.

It is well accepted that social isolation among rodent pups affects brain function, leading the organism to adapt to challenges in the environment. However, the long-term resulting stress can produce maladaptive behaviors and ultimately disease (Marsden et al., 2011; Pereda-Perez et al., 2013). Compared to rodents, social interactions in primates are even more complex. Some years ago it has been proposed that callitrichids when exposed to disturbing conditions do not overreact to it. This response was considered to be due to their familiar organization, which is very complex and stable over time, therefore such familiar relationship could have a buffering effect when these animals are exposed to stressful situations (Rylands, 1993). This however, is in opposition to recent findings (Barbosa and Mota, 2009; Yamaguchi et al., 2010). In spite of their complex organization and stability, in captive conditions families of callitrichids are easily stressed even by their caregivers, showing higher stress levels during week daily care manipulations than during weekends (Barbosa and Mota, 2009). Thus, we believe that individual isolation of juvenile marmosets for 1 or 3 weeks could adequately model anxiety in non-human primates. Age is critical factor as it has been demonstrated that adult marmosets are insensitive to psychosocial stress paradigms that include a recovery period, as measured by neurogenesis in the dentate gyrus (Marlatt et al., 2011).

The potential impact of the reduced neurogenesis reported here is that it is occurring at a critical period, which is essential to make predictions regarding possible future mental disturbances. The transition between childhood and adulthood is characterized by high impulsivity, high plasticity and the development of complex behavioral repertoires, ultimately leading to stable behavioral patterns that guarantee successful survival (Spear, 2000).The transition from adolescence to adulthood varies in different primate species, yet it is well defined and displays many of the characteristic traits described in humans (Pereira and Altmann, 1985; Steinberg et al., 1989). Recent data based on computational estimates suggested that adult-generated neurons could be additionally incorporated to the population of existing (mature) granule cells leading to gradual increases in the total number of neurons (Aimone et al., 2009). Thus, stressing factors, applied during this critical period, as used in our current study may not only affect neuron birth but also the structural organization of the granule cell layer. We do not have data to test this hypothesis thus future studies should address these specific questions using hippocampal-dependent tasks. Our current results demonstrate that rostral hippocampus levels are the most affected in terms of cell proliferation. Similarly it has recently been reported that depressed adult female cynomolgus monkeys also have hippocampal atrophy in rostral but not caudal portions (Willard et al., 2009). It is noteworthy that previous studies consider that posterior hippocampus in humans (or primates) activation may reflect contextual fear encoding, whereas the engagement of rostral regions during later phases of acquisition may reflect the emotional expression of that fear (Bannerman et al., 2003; Fanselow and Dong, 2010).

The greater manifestation of anxiety-related behaviors right after social deprivation was concomitant with increased cortisol levels, as previously demonstrated by others (Smith et al., 1998; Marlatt et al., 2011). Similarly to our results, these authors reported increases in cortisol levels 48 h after isolation with recovery to basal levels when animals had returned to their family. In the current study, past some days of isolation, cortisol slightly decreased until remaining constant between 1 and 3 weeks after familial separation. On the other hand, unlike cortisol levels, anxiety-related behaviors continuously augmented during late phase of isolation. At first, animals that were socially isolated for 1 week showed signs of increased distress with high number of scent marking and high levels of cortisol, similarly to previous findings (Laudenslager et al., 1990, 1995; Norcross and Newman, 1999; Rukstalis and French, 2005). As the social isolation persists, some authors have reported a drop in physiological indicators of stress, indicative of habituation (Honess and Marin, 2006). Our current results on the other hand indicate that even when individual isolation took longer, animals still had a high number of scent marking and locomotor activities, having these anxiety-related behaviors returned to basal levels only after familiar reunion. At the final days in the 1 week isolation group, cortisol level became stable, but not anxiety-like behaviors, indicating here dissociation between hormones and behaviors. Similar findings were described in others studies with different species (Norcross and Newman, 1999; Hennessy et al., 2008) that in turn show the importance of measuring both indicators for more accurate results.

Direct modulation of dentate gyrus cell population by corticoids has been considered an important mechanism through which stressors reduce hippocampal neurogenesis (Mirescu and Gould, 2006). Indeed, already in the early phase of isolation animals had significantly increased cortisol levels, a hormone involved in mediating the effects of stress on neurogenesis. The hippocampus contains glucocorticoid and mineralocorticoid receptors, besides being an important structure in the modulation of stressful responses (for a review see McEwen, 1999). Accordingly, a recent study has demonstrated that early life stress in primate infants leads in adolescence to mild reductions in the expression of mineralocorticoid and glucocorticoid receptor genes in the hippocampus (Arabadzisz et al., 2010). As concluded by these last authors, it is unlikely that these reductions are only acute mediators of the long-term effects of early life stress. Indeed, increases in the corticosterone levels during social isolation in marmosets did alter the number of proliferating cells in the dentate gyrus (Marlatt et al., 2011).

Decreased hippocampal neurogenesis plays a role in depression and anxiety-related behaviors (Wong and Licinio, 2004; Cryan and Holmes, 2005). Nevertheless, it does not establish a direct link between both since inhibition of neurogenesis in mice did not seem to trigger a depressive or anxious behavioral phenotype, but the full capacity of giving rise to new neurons may be crucial for antidepressants to take effect (Santarelli et al., 2003; Balu and Lucki, 2009). Accordingly, as shown here, inhibition of neurogenesis was exacerbated as a time-dependent outcome of social isolation-induced stress, even when cortisol levels were steady (from 1 until 3 weeks of separation). Some reports have indicated that decreased neurogenesis can keep on even after re-establishment of standard cortisol levels (Malberg and Duman, 2003; Mirescu et al., 2004). Thus, the hypothesis that high cortisol levels were not necessary to maintain neurogenesis suppressed is supported by the present results. From this work we can suggest that separation-induced stress affects proliferation of progenitor cells, as well as neuronal fate of newborn cells in the dentate gyrus.

Our current interpretations are limited by the number of animals and current experimental conditions. In rodent brain, profound differences have been reported between males and females in response to early life stress, particularly on neurogenesis (Oomen et al., 2009; Negrigo et al., 2011; Korosi et al., 2012; Lima et al., 2014). In the current study, we showed that gender does not interfere in the dentate gyrus neurogenesis rate (Marlatt et al., 2011), and thus we have chosen to pool male and female animals. Unfortunately, here we could not provide a direct correlation between the cortisol measurements and the dentate proliferating rate given these results were obtained from different sets of animals. Yet, behavioral results did not differ between the two sets of animals and due to the current experimental settings we were able to avoid the death of seven animals.

## Conclusion

In non-human primates, social relationships comprise an important aspect of the juvenile's environment and are crucial for the development of normal behavior. Though these behavioral abnormalities have been somewhat characterized, the mechanisms underlying these events are still elusive. Our data is consistent with the notion that social deprivation leads to stress (indicated by higher cortisol levels), producing anxiety-like behaviors. We showed that some of the consequences of the stressful condition, such as reduction of neurogenesis, have a slower response profile. Our data might be relevant for the understanding of the pathophysiological conditions that ensue after episodes of social separation in humans.

## Authors' contributions

Simone M. Cinini was responsible for animal care at UNIFESP facility, participated in the study design, performed immunohistochemistry, BrdU cell counts, and drafted the manuscript. Gabriela F. Barnabe helped in study design, performed immunofluorescence, carried out confocal microscopy, statistical analysis, and drafted the manuscript. Nicole Galvão-Coelho conducted all behavioral and cortisol measurement experiments and its analysis. Magda A. de Medeiros conceived of the study and helped with animal care. Patrícia Perez-Mendes helped with animal care and participated in the study design. Maria B. C. Sousa designed the behavioral and cortisol experiments, also contributed with data interpretation. Luciene Covolan participated in experimental design, conducted cell count acquisition and interpretation, drafted the manuscript. Luiz E. Mello conceived of the study, and participated in its design and coordination and helped to draft the manuscript. All authors read and approved the final manuscript.

### Conflict of interest statement

The authors declare that the research was conducted in the absence of any commercial or financial relationships that could be construed as a potential conflict of interest.

## Abbreviations

1W: socially isolated for one week
3W: socially isolated for three weeks
BrdU: 5-bromo-2′-deoxyuridine
PBS: phosphate buffered saline
DAB: 3,3′-diaminobenzidine-tetrahydrochloride
DCX: doublecortin.
